# Influence of Semiquantitative [^18^F]FDG PET and Hematological Parameters on Survival in HNSCC Patients Using Neural Network Analysis

**DOI:** 10.3390/ph15020224

**Published:** 2022-02-14

**Authors:** Paulina Cegla, Geoffrey Currie, Joanna P. Wróblewska, Witold Cholewiński, Joanna Kaźmierska, Andrzej Marszałek, Anna Kubiak, Pawel Golusinski, Wojciech Golusiński, Ewa Majchrzak

**Affiliations:** 1 Department of Nuclear Medicine, Greater Poland Cancer Center, 61-866 Poznan, Poland; witold.cholewinski@wco.pl; 2 School of Dentistry and Health Science, Charles Sturt University, Wagga Wagga 2678, Australia; gcurrie@csu.edu.au; 3 Department of Oncologic Pathology and Prophylaxis, Poznan University of Medical Sciences, 61-701 Poznan, Poland; joanna.wroblewska@wco.pl (J.P.W.); andrzej.marszalek@wco.pl (A.M.); 4 Department of Tumor Pathology, Greater Poland Cancer Centre, 61-866 Poznan, Poland; 5 Department of Electroradiology, Poznan University of Medical Science, 61-701 Poznan, Poland; joanna.kazmierska@wco.pl; 6 2nd Radiotherapy Department, Greater Poland Cancer Center, 61-866 Poznan, Poland; 7 Greater Poland Cancer Registry, Greater Poland Cancer Centre, 61-866 Poznan, Poland; anna.kubiak@wco.pl; 8 Department of Otolaryngology and Maxillofacial Surgery, University of Zielona Gora, 65-046 Zielona Góra, Poland; pgolusinski@uz.zgora.pl; 9 Department of Head and Neck Surgery, Poznan University of Medical Sciences, Greater Poland Cancer Center, 61-866 Poznan, Poland; wojciech.golusinski@wco.pl (W.G.); ewa.majchrzak@wco.pl (E.M.)

**Keywords:** positron emission tomography/computed tomography, head and neck squamous cell carcinoma, overall survival, neural network

## Abstract

The aim of this study is to assess the influence of semiquantitative PET-derived parameters as well as hematological parameters in overall survival in HNSCC patients using neural network analysis. Retrospective analysis was performed on 106 previously untreated HNSCC patients. Several PET-derived parameters (SUV_max_, SUV_mean_, TotalSUV, MTV, TLG, TLR_max_, TLR_mean_, TLR_TLG_, and HI) for primary tumor and lymph node with highest activity were assessed. Additionally, hematological parameters (LEU, LEU%, NEU, NEU%, MON, MON%, PLT, PLT%, NRL, and LMR) were also assessed. Patients were divided according to the diagnosis into the good and bad group. The data were evaluated using an artificial neural network (Neural Analyzer version 2.9.5) and conventional statistic. Statistically significant differences in PET-derived parameters in 5-year survival rate between group of patients with worse prognosis and good prognosis were shown in primary tumor SUV_max_ (10.0 vs. 7.7; *p* = 0.040), SUV_mean_ (5.4 vs. 4.4; *p* = 0.047), MTV (23.2 vs. 14.5; *p* = 0.010), and TLG (155.0 vs. 87.5; *p* = 0.05), and mean liver TLG (27.8 vs. 30.4; *p* = 0.031), TLR_max_ (3.8 vs. 2.6; *p* = 0.019), TLR_mean_ (2.8 vs. 1.9; *p* = 0.018), and in TLR_TLG_ (5.6 vs. 2.3; *p* = 0.042). From hematological parameters, only LMR showed significant differences (2.5 vs. 3.2; *p* = 0.009). Final neural network showed that for ages above 60, primary tumors SUV_max_, TotalSUV, MTV, TLG, TLR_max_, and TLR_mean_ over (9.7, 2255, 20.6, 145, 3.6, 2.6, respectively) are associated with worse survival. Our study shows that the neural network could serve as a supplement to PET-derived parameters and is helpful in finding prognostic parameters for overall survival in HNSCC.

## 1. Introduction

Head and neck squamous cell carcinoma (HNSCC) originally develop from the mucosal epithelium in the oral cavity, pharynx, and larynx and is the sixth most common cancer worldwide, with 890,000 new cases and 450,000 deaths in 2018 [1]. Most oral cavity and larynx tumors are associated with alcohol and tobacco intake, while oropharynx are linked with human papilloma virus (HPV) infection [2]. Thus, the recent TNM classification mentioned the differences between HPV-positive and HPV-negative patients diagnosed with oropharyngeal cancer [3]. The 5-year survival rate for patients with localized disease is approximately 80%; around 50% in cases with lymph nodes metastases and 20% when distant metastases are diagnosed [4].

Positron emission tomography combined with computed tomography (PET/CT) is a commonly used imaging method in oncological patients. With the most widely used radiotracer 2-deoxy-2-[^18^F]fluoro-D-glucose ([^18^F]FDG), the method provides several metabolic and volumetric parameters such as standardized uptake value (SUV), metabolic tumor volume (MTV), total lesion glycolysis (TLG), and tumor-to-liver ratio (TLR), which are useful in assessing recurrence and overall survival (OS) in cancer patients including HNSCC [5,6,7].

In clinic, inflammatory markers including neutrophil-to-lymphocyte ration (NRL), platelet-to-lymphocyte ratio (PLR), monocyte-to-lymphocyte ratio (MLR), and lymphocyte-to-monocyte ratio (LMR) have been reported as prognostic factors in several solid tumors including breast cancer, colorectal cancer, lung cancer, prostate cancer, ovarian cancer, and HNSCC [8,9,10,11,12,13]. Based on these results, inflammatory response biomarkers are considered to be an additional tool for predicting outcomes in cancer patients [13,14].

Artificial Intelligence (AI) has begun to play an important role in almost all industries as well as in medical imaging. Machine learning (ML) has been used to predict subtypes of disease as it learns from the experience (medical images) and PET-based AI imaging helps to assess the clinical decision [15]. It is used in head and neck cancer for prediction of diagnosis, treatment response, overall survival, and defining gross tumor volume (GTV) [16,17].

**Figure 1:**
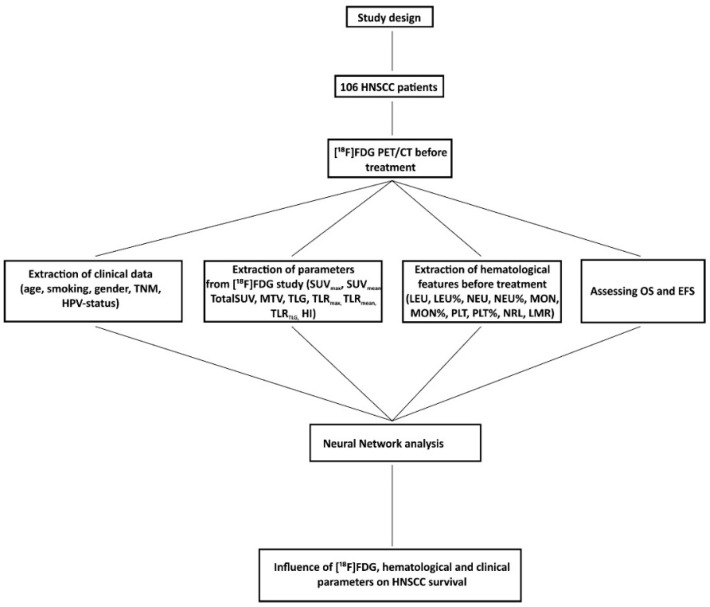
Overview diagram.

The objective of this study is to assess the influence of PET-derived and hematological parameters on survival in head and neck squamous cell carcinoma patients using neural network analysis (Figure 1).

## 2. Materials and Methods

### 2.1. Patient Characteristics

A medical chart review was made and data related to age at the time of diagnosis, prior or current history of smoking, gender, TNM stage, HPV-status, and patient treatment were determined. Data obtained from the Greater Poland Cancer Registry Poznan, Poland were used to estimate the patients’ prognosis; based on these data, patients were divided into two groups: with good prognosis (they are still alive) and with worse prognosis (in whom cancer-related death was confirmed). Survival was assessed from date of primary diagnosis to death or date of the last information. Follow-up was assessed from the primary diagnosis to the date of the last information or death with mean time of 60 ± 25 months (ranged from 18–96 months). Patients were assessed during their clinical follow-ups for the first year every month, for the second year every 2 months, in the third year every three months, and every six months thereafter.

### 2.2. [^18^F]FDG PET/CT Analysis

**Figure 2:**
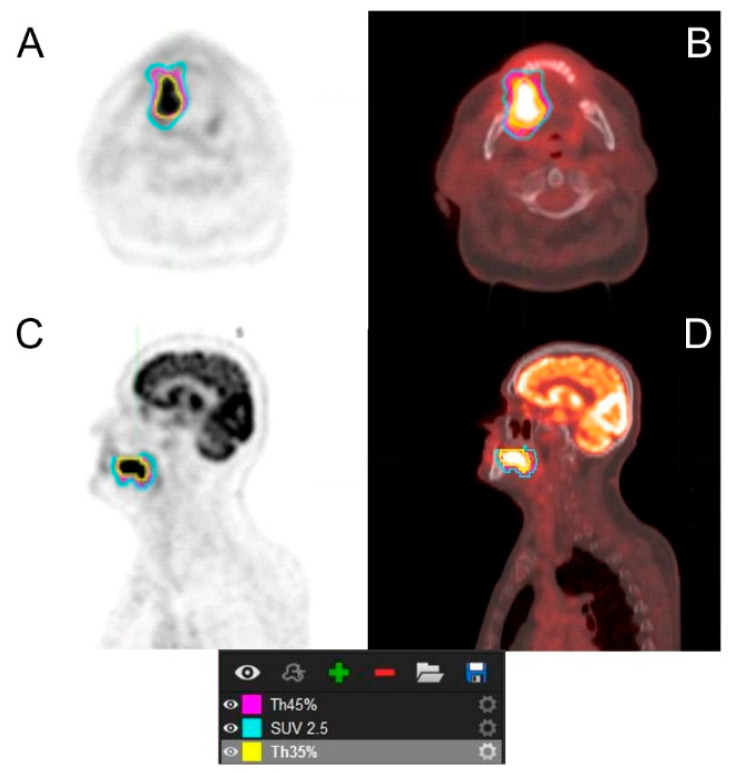
An example of segmented image according to the chosen segmentation method. (**A**) PET images in transverse plane, (**B**) fused PET/CT images in transverse plane, (**C**) PET image in sagittal plane, (**D**) fused PET/CT images in sagittal plane.

Retrospective single-center analysis was performed on a group of histologically confirmed 106 HNSCC patients in whom a pretreatment [^18^F]FDG PET/CT study was performed between June 2009 and December 2019. PET scans were acquired with a Gemini TF PET/CT scanner 60–75 min p.i. 364 ± 75 MBq of [^18^F]FDG according to the standard EANM protocols to avoid artifacts and poor quality of images [18]. The review of fused PET/CT images and voxel measurements of the tumor were performed on a dedicated workstation in sagittal, coronal, and transverse planes. Choosing an appropriate contour method is crucial because it has an influence on the values of obtained parameters, especially SUV_mean_, MTV, and TLG. According to our previous study based on phantom analysis, Th35% was used as an appropriate segmentation method for tumor and lymph nodes delineation (Figure 2) [19].

**Figure 3:**
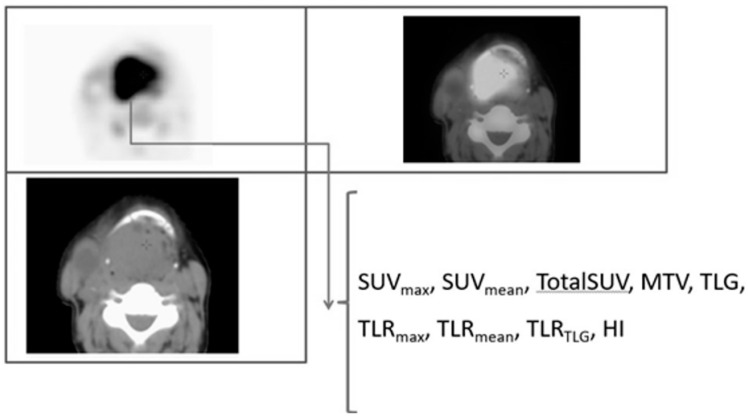
[^18^F]FDG PET-derived assessed parameters.

Several semiquantitative PET parameters including SUV_max_, SUV_mean_, TotalSUV, MTV, TLG, TLR_max_, TLR_mean_, TLR_TLG_, and heterogeneity index (HI) for primary tumor (Figure 3) and lymph node with highest activity were assessed.

TLG was calculated as a product of the SUV_mean_ and the MTV. The determination of liver SUV was obtained by drawing a 3D spherical ROI with a diameter of 3 cm in the normal right lobe of the liver. TLR_max_ was defined as the ratio of primary tumor SUV_max_ to individual liver SUV_max_. Similar TLR_mean_ and TLR_TLG_, were calculated. HI was estimated according to the following formula: HI=SUV_max_/SUV_mean_ [20]. Moreover, maximum, mean, and TLG lymph-nodes-to-tumor status (LN/T) were assessed. Statistical significance was calculated using Chi-Square analysis for nominal data and Student’s t test for continuous data. A p value less than or equal to 0.05 was considered significant.

### 2.3. Hematological Parameters Analysis

Hematological parameters such as absolute and percentage lymphocyte count (LEU, LEU%), absolute and percentage neutrophil count (NEU, NEU%), absolute and percentage monocyte count (MON, MON%), absolute and percentage platelet count (PLT, PLT%), neutrophil-to-lymphocyte ratio (NRL), and lymphocyte-to-monocyte ratio (LMR) were assessed within 3 days before treatment. Mean time between [^18^F]FDG PET/CT study and the treatment was one month (ranged from 2–5 months). Patients with the sign of infection were excluded from the analysis.

### 2.4. Neural Network Analysis

The data were evaluated using an artificial neural network developed using the in situ computer program previously described [21] (Neural Analyser version 2.9.5). There were 57 available input variables in 106 patients using a binary classification of better than median survival or worse than median survival. The heat map/correlation matrix identified redundant variables omitted from the neural network architecture. A 60:20:20 instances (cases) split was used for training, selection, and testing, respectively. The network architecture included 49 scaling layer inputs and 3 hidden layers of 6 nodes each, using a logistic activation function for a single probabilistic layer (binary). The weighted squared error method was used to determine the loss index and the neural parameters norm was used for the regularization method. A Quasi-Newton training method was employed using gradient information to estimate the inverse Hessian for each iteration of the algorithm (no second derivatives). The loss function associated with the training phase estimates the error associated with the data the neural network observes. The selection loss is a measure of the neural network’s agility and generalizability to new data. The initial value of the training loss was 1.198 and the final value was 0.0157 after 166 iterations. The initial value of the selection loss was 2.413 and the final value was 2.402 after 166 iterations. Detailed explanations and insights into neural network function and architecture have previously been published [22,23].

## 3. Results

The study included 106 patients of whom 28.3% (*n* = 30) were female and 77.7% (*n* = 76) were male. The most common tumor localization was oropharynx 48.1% (*n* = 51) and hypopharynx/larynx 21.7% (*n* = 23), followed by patients with CUP Syndrome 15.1% (*n* = 16) and oral cavity 14.2% (*n* = 15). In one patient, the tumor was localized in nasopharynx (1%). Among all patients, 70 were smokers while 36 were not. Moreover, 28 patients were HPV-positive, while the remaining 78 patients were HPV-negative. The majority of patients (*n* = 100) were diagnosed in M0 stage, while M1 was diagnosed in 6 patients.

Significant differences in assessed parameters based on a patient’s T stage are shown in Table 1.

### 3.1. Differences in [^18^F]FDG Parameters

Patients with N3 stage had significantly higher (*p* < 0.001) primary tumor MTV (48.4) and TLG (471.1) values compared with those with N1 stage (12.3 and 69.8, respectively) and N2 stage (15.5 and 80.6, respectively).

Lymph node SUV_max_ and SUV_mean_ values were significantly higher (*p* = 0.022 and *p* = 0.036) in patients with N3 stage (10.6 and 5.7) compared with those with N1 (4.9 and 2.5) and N2 stages (6.4 and 3.7, respectively). Further, TLR_TLG_ value showed significantly higher values (*p* = 0.002) in N3 stage compared with N1 and N2 (14.1 vs. 3.0 and 2.9, respectively).

Patients with CUP Syndrome had significantly higher (*p* < 0.001) values of Total SUV, MTV, TLG, and TLR_TLG_ compared with patients with other T1–T4 stages (Table 2).

From hematological parameters, only PLT appeared to differ significantly between CUP Syndrome patients and patients with T4 stage of the primary tumor (207.3 vs. 287.3, *p* = 0.027).

No other statistically significant differences were shown between analyzed hematological and PET-derived parameters.

### 3.2. Overall Survival Analysis

**Figure 4:**
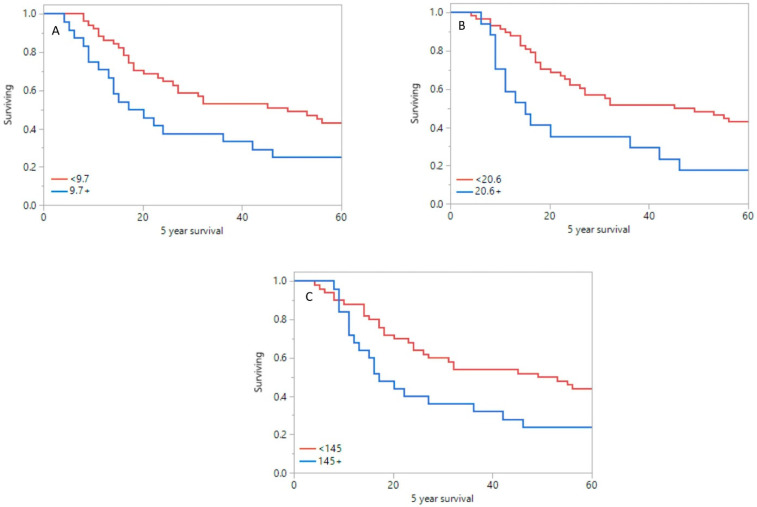
Survival plot for primary tumor SUV_max_ (**A**), MTV (**B**), and TLG (**C**).

Mean time for the whole group was 24 ± 18 months and 58 ± 34 months for OS and EFS, respectively. Statistically significant differences in 5-year survival rate between group of patients with worse prognosis and good prognosis were shown in primary tumor SUV_max_ (10.0 vs. 7.7; *p* = 0.040, Figure 4A), SUV_mean_ (5.4 vs. 4.4; *p* = 0.047), MTV (23.2 vs. 14.5; *p* = 0.010, Figure 4B), and TLG (155.0 vs. 87.5; *p* = 0.05, Figure 4C). In lymph nodes, significant differences in 5-year survival between group with bad and good prognosis were shown in SUV_max_ (7.8 vs. 6.6; *p* = 0.006) and SUV_mean_ values (4.2 vs. 3.74; *p* = 0.007).

**Figure 5:**
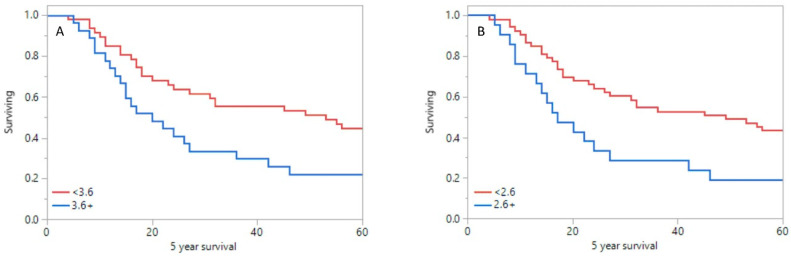
Survival plot for TLR_max_ (**A**) and TLR_mean_ (**B**).

Moreover, significant differences between patients with bad and good prognosis were shown in mean liver TLG (27.8 vs. 30.4; *p* = 0.031), TLR_max_ (3.8 vs. 2.6; *p* = 0.019, Figure 5A), TLR_mean_ (2.8 vs. 1.9; *p* = 0.018, Figure 5B), and TLR_TLG_ (5.6 vs. 2.3; *p* = 0.042).

From hematological parameters, only LMR showed significant differences in 5-year survival between patients who are dead and who are still alive (2.5 vs. 3.2; *p* = 0.009).

Comparing 5-year survival rate in HNSCC patients showed significant differences between smokers and nonsmokers (39.3 vs. 65.1, *p* = 0.006), HPV-positive and HPV-negative status (75.2 vs. 37.8, *p* < 0.001), and between patients with tumor in oropharynx and CUP Syndrome (57.1 vs. 21.3, *p* = 0.031). Moreover, patients with CUP Syndrome had a significantly worse (*p* = 0.001) 5-year survival rate compared with patients with T1 (21.1 vs. 89.7), T2 (21.1 vs. 47.2), T3 (21.1 vs. 42.4), and T4 (21.1 vs. 43.8) stage.

### 3.3. Neural Network Analysis

**Figure 6:**
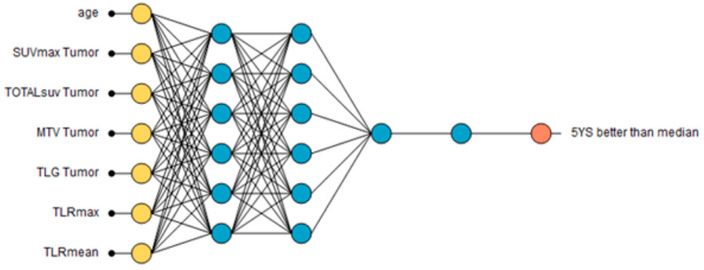
Final architecture of the neural network.

A growing inputs method was used to calculate the correlation for every input against each output in the data set. Beginning with the most highly correlated inputs, progressively decreasing correlated inputs were added to the network until the selection loss increased. The final architecture of the neural network reflects the optimized subset of inputs with the lowest selection loss. In this case, the selection loss and the training loss identified the optimal number of inputs to be 7 following 9 iterations. The final architecture was 7 scaling layer inputs (yellow); 3 hidden layers (blue) of 6; 6 and 1 nodes, respectively; and a single binary probabilistic layer (red) (Figure 6).

Receiver operator characteristic (ROC) analysis showed an area under the curve of 0.905 with high sensitivity of 0.889 and specificity of 0.857 for predicting greater than median survival. Classification accuracy was 0.625, precision 0.8, F1 score 0.571, Matthews correlation 0.323, and Youden index 0.302. Maximum gain score was 0.746 at 0.6 instances ratio. Lower than median survival is predicted by the following:

- Age 60+ years;
- SUV_max_ tumor over 9.7;
- TotalSUV tumor over 2255;
- MTV tumor over 20.6;
- TLG tumor over 145;
- TLR_max_ over 3.6;
- TLR_mean_ over 2.6.

## 4. Discussion

It has been reported that an [^18^F]FDG PET/CT study is a better imaging method than other diagnostic modalities in staging and assessing the recurrence in HNSCC patients [24]. Several studies reported that, in HNSCC patients, the SUV_max_ value is a prognostic factor of survival regardless of the tumor size, however, with no specific cut-off value ranging from 4–10 [25,26]. Querellou et al. investigated a group of 89 HNSCC patients and showed that the best cut-off of primary tumor for disease-free survival (DFS) and OS is 7 [27]. Minn et al., in a multivariate analysis, showed that baseline SUV_max_ of primary tumor with cut-off of 9 showed significant differences in OS in HNSCC patients. For patients with SUV_max_ less than 9.0, the 3-year DFS was 54% compared with 24% for patients with primary tumor SUV_max_ greater than 9 [28]. In our study, we confirmed these results and showed that patients with higher SUV_max_ (above 9.7) and SUV_mean_ of the primary and higher SUV_max_ of the lymph nodes (above 7.0) showed significant differences between 5-year survival rates. Even so, SUV_max_ cut-off values for primary tumor varied in different studies. Paidpally et al. suggested that patients with SUV_max_ higher than 9 have worse OS and progression-free survival (PFS), regardless of the heterogeneity of the therapy [29], which is in accordance with our results, based on a neural network analysis. We showed that SUV_max_ of primary tumor greater than 9.7 is one of the parameters associated with worse survival rate.

Torizuka et al., analyzed 50 head and neck cancer patients and showed that primary tumor SUV_max_ correlates with T stage and N stage [30]. Scott et al., similar to our analysis, assessed SUV_max_, MTV, and TLG for primary tumor and for the most active lymph node. In the univariate analysis, they showed that primary tumor SUV_mean_ and SUV_max_ of the most active node and nodal TLG were significant predictors for OS in oral cavity cancer patients. Moreover, from demographic parameters, only age significantly predicts OS in these patients [31]. Contrary to others, our analysis suggests that from all assessed metabolic parameters, only SUV_max_ and SUV_mean_ of the hottest lymph node showed a significant difference in N3 stage compared with other N-stages. No significant differences or correlations were found between SUV_max_ or SUV_mean_ of the primary tumor and T-stage. However, in the neural network analysis, age above 60 was one of the predictors that correlated with worse prognosis.

Contrarily, other authors have suggested that MTV of the primary tumor is a prognostic imaging biomarker in several solid tumors including lung, esophageal, and ovarian cancer [32,33,34]. Abgral et al., in their study on 80 HNSCC patients, showed that MTV greater than 4.86 is an independent prognostic factor for OS and event-free survival (EFS); moreover, the authors suggested that in patients with this MTV value, more aggressive treatment or close monitoring should be included [35]. La et al. reported that a preradiation increase in MTV of 17.4 mL was significantly associated with a 1.9-fold increase in recurrence and 2.1-fold increase in death [36]. Tang et al. showed that total MTV greater than 17 correlated with 2.1-fold of progression, with 2-fold risk of death; in further analysis, it was shown that total MTV was due to tumor MTV, while nodal MTV did not show any significance in PFS or OS [37]. Similar to others, our study confirmed the abovementioned statements that a higher MTV of primary tumor is associated with worse prognosis in HNSCC patients. Additionally, neural network showed that a MTV cut-off of primary tumor greater than 20.6 correlates with 5-year survival in HNSCC.

Another volumetric parameter which is of great interest in assessing overall survival is TLG. It has been shown that this parameter demonstrates a prognostic value in lung, breast, and rectal cancer [38,39,40]. Cheng et al. assessed retrospectively [^18^F]FDG PET images of 60 OPSCC patients with determined HPV status. They found that HPV-positive and high primary tumor TLG (with cut-off value of 135.3) were significantly associated with OS, whereas only primary tumor TLG was an independent prognostic factor for DFS, PFS, and locoregional control [41]. Moon et al. concluded that TLG is the only significant predictor for OS in patients with SCC of the tonsil [42]. Similarly to studies mentioned above, our analysis has shown that both HPV-positive and primary tumor TLG have an influence on 5-year overall survival in HNSCC. Moreover, we have also shown that mean liver TLG and TLR_TLG_ are significantly different between patients with good and worse prognosis.

The liver uptake is one of the most widely used reference backgrounds because of a low change in uptake time after the radiotracer injection. Elevated tumor-to-liver ratio has been reported as a predictor in various cancers including lymphoma, lung, and colorectal cancer [7,43,44]. Recently Choi et al. analyzed several PET parameters (including SUV_max_, TLR, MTV, and TLG) in primary tumor and lymph nodes on OS and DFS in patients with oropharyngeal cancer [45]; however, they did not find any relationship between TLR and OS or DFS. Our results suggest that patients with higher TLG_max_, TLG_mean_, and TLG_TLR_ have worse OS. Moreover, based on a neural network analysis, we showed that TLG_max_ over 3.6 and TLR_mean_ over 2.6 were associated with worse prognosis for patients with HNSCC.

Some studies suggest that hematological parameters have prognostic value in several cancers [46,47]. Recently, Ohashi et al. showed that [^18^F]FDG PET/CT study has the potential to reflect cancer-related chronic inflammation in HNSCC patients [48]. Guo et al. concluded that incorporation of NRL and SUV_max_ improves prediction of clinical outcome in patients with locally advanced non-small-cell lung cancer [49]. In this study, we evaluated several hematological parameters and analyzed their correlation with OS in HNSCC. Among analyzed parameters, LMR showed a significant difference in 5-year survival rate. Additionally, PLT count significantly differed between patients with CUP Syndrome and those with T4-stage of the primary tumor.

It should be mentioned that this study has some limitations. Firstly, this was a single-center and retrospective analysis on a heterogeneous (based on primary tumor localization) group of HNSCC patients. Secondly, the implemented treatment methods were not homogenous, as they were adjusted to the clinical stage of the patients. To overcome these limitations, a larger multicenter study should be performed in the future to confirm the results of our preliminary study.

## 5. Conclusions

Our study shows that neural network could serve as a supplement to PET-derived parameters and is helpful in finding prognostic parameters for overall survival in HNSCC. Combining clinical well-known factors such as stage of the disease, primary tumor site, and HPV status, with [^18^F]FDG PET-derived primary tumor parameters such as SUV_max_, TotalSUV, MTV, TLG, and TLR_max_, TLR_mean_ and age over 60 can provide additional helpful information and might assist in more accurate risk stratification of HNSCC patients. Further studies with a more homogenous group of patients in terms of tumor localization are planned.

## Figures and Tables

**Table 1:** Summary of data by T and N stage.

T Stage	TX	T1	T2	T3	T4	*P*
Patient details						
Proportion of studies (%)	16 (13.5)	18 (16.4)	18 (16.4)	19 (18.3)	35 (35.6)	-
Mean age (years)	63.4	53.8	55.8	59.0	56.7	0.098
Male (%)	78.6	76.5	64.7	68.4	70.3	0.901
Smoker (%)	64.3	52.9	70.6	68.4	64.9	0.696
Mean packs/year	13.1	12.8	17.7	17.1	16.9	0.871
Mean overall survival (months)	14.4	62.7	18.0	22.6	25.1	0.003
Mean event free survival (months)	31.0	68.6	62.4	48.5	66.3	0.156
HPV+ (%)	0	58.8	47.1	31.6	10.8	<0.001
Tumor localization %						
Hypopharynx/larynx	0	5.9	29.4	15.8	37.8	<0.001
Nasopharynx	0	0	0	0	2.7	
Oropharynx	0	88.2	52.9	68.4	35.1	
Oral cavity	0	5.9	17.7	10.3	21.6	
CUP	100	0	0	0	0	
Differentiation						
G1	0	5.9	5.9	0	5.4	0.006
G2	7.1	58.8	64.7	68.4	67.6	
G3	35.7	17.7	17.7	26.3	16.2	
N staging						
0	0	12.5	25.0	26.3	13.9	0.004
1	0	18.8	12.5	0	8.3	
2	50	56.3	56.3	63.2	75	
3	50	12.5	6.3	10.5	2.8	
M0 stage (%)	15.6	19.5	19.5	23.4	22.1	0.271
Treatment						
Surgery	0	0	11.8	0	0	0.001
Chemotherapy	7.1	0	0	0	0	
RT	21.4	0	11.8	26.3	8.1	
Surgery/Chemo	14.3	5.9	5.9	0	0	
Surgery/RT	42.9	35.3	11.8	5.3	8.1	
Surgery/Chemo/RT	7.1	35.3	29.4	31.6	24.3	
RTCH	7.1	23.5	29.4	36.8	59.5	

Abbreviations: HPV+—human papillomavirus positive patients; CUP—cancer of unknown primary; RT—radiotherapy; chemo—chemotherapy; RTCH—radio-chemotherapy; TX—patients with unknown primary (CUP); T1—patients with T1 stage; T2—patients with T2 stage; T3—patients with T3 stage; T4—patients with T4 stage; *P*—*p* value.

**Table 2:** PET parameters assessed in CUP patients and other T-stages.

Parameter	CUP	T1	T2	T3	T4
TotalSUV	6984.1	307.5	850.1	1396.2	1519.8
MTV (cm^3^)	47.1	6.0	12.0	17.0	18.1
TLG	447.0	20.8	54.4	86.7	98.7
TLR_TLG_	13.3	0.7	2.1	2.6	3.8

Abbreviations: CUP—Cancer with Unknown Primary; T1–T4—patients with T1–T4 stage of primary tumor; MTV—metabolic tumor volume; TLG—total lesion glycolysis; TLR_TLG_—tumor-to-liver total lesion glycolysis ratio.

## Data Availability

The data presented in this study are available on request from the corresponding author. The data are not publicly available due to legal requirements of data protection.
